# Development and validation of a multiphase CT radiomics-based machine learning model for prognostic prediction in sarcomatoid renal cell carcinoma

**DOI:** 10.3389/fonc.2026.1982796

**Published:** 2026-10-30

**Authors:** Tongpeng Liu, Yu Yao, Zhijian Zhou, Yang Hu, Lijiang Sun, Guiming Zhang

**Affiliations:** 1 Department of Urology, The Affiliated Hospital of Qingdao University, Qingdao, China; 2 Department of Urology, School of Rehabilitation of Capital Medical University, China Rehabilitation Research Center, Beijing, China; 3 Department of Radiology, The Second People’s Hospital of Chengyang District, Qingdao, China

**Keywords:** machine learning, multiphase CT, nomogram, prognostic model, radiomics, sarcomatoid renal cell carcinoma

## Abstract

**Background:**

Sarcomatoid renal cell carcinoma (sRCC) is a rare and aggressive subtype of renal cell carcinoma with a poor prognosis. Accurate prognostic stratification is critical for individualized treatment planning. This study aimed to develop and validate a machine learning-based prognostic model integrating multiphase CT radiomics and clinicopathological factors to predict overall survival (OS) in patients with pathology-confirmed sRCC.

**Methods:**

A total of 113 patients with pathologically confirmed sRCC who underwent nephrectomy were retrospectively enrolled. Radiomic features were extracted from arterial phase (AP) and venous phase (VP) CT images. After feature selection using intraclass correlation coefficient (ICC > 0.75) and univariate Cox regression, a radiomics signature was constructed using least absolute shrinkage and selection operator (Lasso) regression and random survival forest (RSF) algorithms. A combined model was developed by integrating the radiomics score with independent clinical predictors identified through multivariate Cox regression. Model performance was evaluated using the concordance index (C-index), time-dependent receiver operating characteristic (ROC) curves, calibration curves, and decision curve analysis (DCA). Internal validation was performed using bootstrap resampling and repeated cross-validation.

**Results:**

The combined model, incorporating the AP+VP radiomics score, N stage, M stage, and Ki-67 index, showed numerically improved predictive performance compared with the clinical model. In the held-out test set, it achieved a C-index of 0.737 (95% CI: 0.612–0.846), which was comparable to the AP+VP radiomics model alone (C-index: 0.735) and numerically higher than the clinical model (C-index: 0.615); however, the improvement over the clinical model was not statistically significant (P = 0.115). AUCs for 1-, 2-, and 3-year OS were 0.833, 0.780, and 0.749, respectively. The model showed good calibration and clinical utility across a range of threshold probabilities. Significant survival stratification was achieved between high- and low-risk groups (log-rank P < 0.05).

**Conclusions:**

The proposed machine learning model combining multiphase CT radiomics and clinicopathological factors may provide a noninvasive tool for predicting OS in sRCC patients. It showed numerically improved performance compared with conventional clinical models; however, this incremental benefit was not statistically significant, and its performance was comparable to the radiomics-only model. Further multicenter validation is warranted to confirm its generalizability.

## Introduction

Sarcomatoid renal cell carcinoma (sRCC) is a rare and highly aggressive subtype of renal cell carcinoma, accounting for approximately 1–5% of all RCC cases (1, 2). It is characterized by a high propensity for early metastasis and marked chemoresistance, conferring a poor prognosis with a median overall survival typically of less than 12 months (1, 2). Epidemiological studies indicate that the proportion of sRCC among all RCC cases increased between 2010 and 2019, accompanied by a rising incidence of stage I disease (3). Currently, radical nephrectomy remains the standard of care for localized sRCC; however, clinical observations indicate that more than 50% of patients experience disease recurrence or distant metastasis after surgery (4). Given the poor prognosis associated with sRCC, accurate prognostic models are of considerable clinical importance for guiding individualized treatment strategies and improving patient outcomes.

The TNM staging system, established by the American Joint Committee on Cancer (AJCC), remains the gold standard for prognostic assessment in sRCC (5). However, clinical studies have revealed considerable survival heterogeneity among patients with sRCC within the same TNM stage, underscoring the limitations of a purely anatomy-based staging system for outcome prediction. In recent years, increasing attention has been paid to the role of host microenvironmental factors, particularly systemic inflammatory responses, in sRCC progression. Inflammatory markers such as the Systemic Inflammation Response Index (SIRI) have been shown to correlate with poor prognosis in RCC by mediating tumor microenvironment remodeling, promoting angiogenesis, and facilitating immune evasion (6–8). Notably, sRCC exhibits substantial intratumoral heterogeneity, which poses challenges for conventional imaging in comprehensively assessing its biological behavior. Radiomics has emerged as a promising approach to address this issue. This technique enables noninvasive quantification of tumor heterogeneity through high-throughput extraction of quantitative features—including texture and wavelet features—from computed tomography (CT) images (9, 10). Similarly, Alhussaini et al. applied radiomics and machine learning methods to clear cell RCC (ccRCC) to predict tumor grading by analyzing intratumoral regional heterogeneity, further demonstrating the potential of this technology for characterizing heterogeneity in renal cancer (11). As a routine imaging modality for renal cancer diagnosis, multiphase contrast-enhanced CT (CECT) accurately depicts tumor hemodynamics, making it an ideal data source for radiomic analysis (12, 13).

Machine learning (ML) algorithms have garnered considerable interest in medical research because of their strong performance in integrating multimodal data and constructing high-dimensional predictive models. In the context of healthcare big data, the application of ML to disease risk prediction has become an important research focus (14). Notably, the integration of ML with radiomics has demonstrated substantial clinical value in cancer prognosis prediction, offering a precise, dynamic, and noninvasive approach to individualized care (15). Existing studies have confirmed the robust predictive performance of radiomics-based prognostic models across various malignancies, including hepatocellular carcinoma (16), breast cancer (17), and colorectal cancer (18). However, despite the widespread application of ML and radiomics in prognostic research, no systematic studies have yet explored their use for outcome prediction in sRCC. Therefore, comparing the performance of different ML models in radiomics-based prognosis prediction for sRCC may provide a valuable theoretical basis and clinical reference.

This study aims to evaluate the application of multiple ML algorithms in building prognostic models for sRCC based on CECT-derived radiomic features, and to develop an integrated prediction model incorporating clinical characteristics. The development and reporting of the model were guided by contemporary recommendations for clinical prediction models (19, 20). We hope to provide an accurate, noninvasive, and effective novel method for prognostic assessment in patients with sRCC, thereby supporting clinical decision-making.

## Methods

### Patients

This study was conducted in strict accordance with the ethical principles of the Declaration of Helsinki (2013 revision) and was approved by the Institutional Ethics Review Board (Approval No. QYFYWZLL30032). As this was a retrospective analysis, the requirement for informed consent was waived. Patients who underwent radical surgery and had histopathologically confirmed sRCC at the Affiliated Hospital of Qingdao University between December 2009 and September 2024 were included. The inclusion criteria were as follows: (1) postoperative pathological confirmation of sRCC with complete clinical data; and (2) CECT performed within 2 weeks before surgery. Exclusion criteria were: (1) missing clinical or pathological data; (2) severe CT artifacts affecting radiomic analysis; (3) unavailable CECT images that met quality requirements; or (4) receipt of neoadjuvant therapy. To ensure objectivity, two independent researchers screened all potential cases using uniform standards. Any discrepancies were resolved by a third senior researcher until consensus was achieved. A total of 113 eligible patients with sRCC were included and randomly assigned at a 2:1 ratio to a training cohort (n = 75) or a held-out test set (n = 38) using computer-based randomization. The patient screening process is summarized in **Figure 1**. Clinical data included demographic characteristics (sex and age) and laboratory parameters (complete blood count, biochemical indicators, etc.). For variables with <5% missing values, mean imputation was applied.

**Figure 1:**
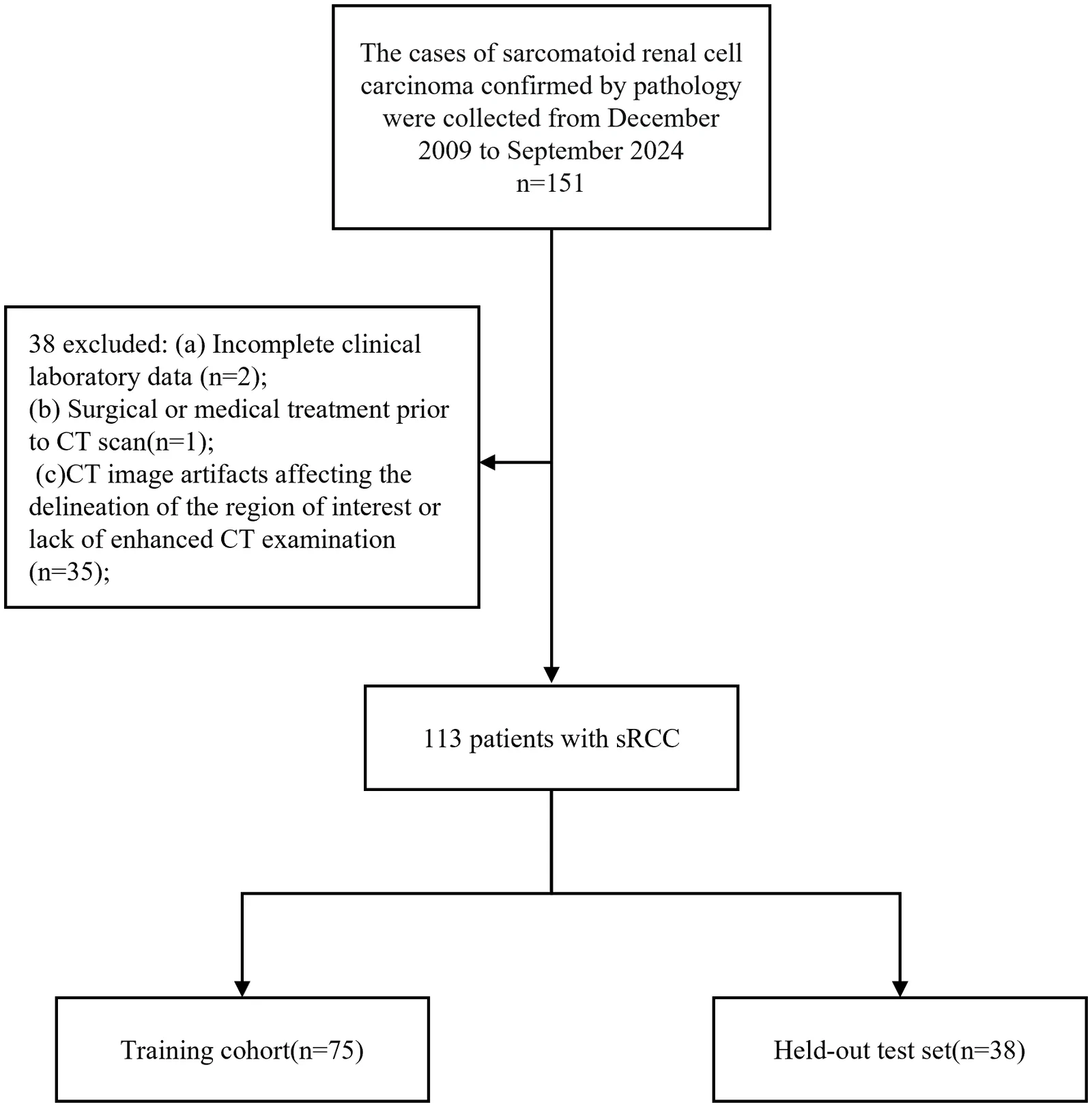
Flow diagram depicting the patient selection process.

### Training and validation strategy

Patients were randomly assigned at a 2:1 ratio to the training cohort (n = 75) and the held-out test set (n = 38). The training cohort was used exclusively for all outcome-dependent feature selection, model construction, algorithm comparison, hyperparameter tuning, and nomogram development. The held-out test set remained untouched until final evaluation and was not used for normalization parameter estimation, feature filtering, univariable or multivariable Cox regression, radiomics score construction, algorithm selection, hyperparameter optimization, nomogram development, or cutoff determination. The final model was locked before it was applied to the held-out test set.

### Clinical data collection and follow-up

Clinical data were retrieved from the hospital’s electronic medical record system and included demographic characteristics (age and sex), body mass index (BMI), TNM stage, complete blood count, and biochemical test results. Inflammatory and other laboratory parameters were calculated from these hematological values. The Ki-67 index was obtained from postoperative pathology reports. Ki-67 was assessed by immunohistochemistry on formalin-fixed paraffin-embedded tumor sections according to standard institutional pathology protocols. The Ki-67 index was defined as the percentage of tumor cells with positive nuclear staining and was recorded as a proportion (0–1). The specific antibody clone and scoring details could not be uniformly retrieved from the retrospective pathology records; this is acknowledged as a limitation. Ki-67 was treated as a continuous variable in the primary analysis rather than being dichotomized by a prespecified cutoff.

The primary endpoint was overall survival (OS), defined as the time from pathological diagnosis to death from any cause or to the last follow-up. Patients who were alive or lost to follow-up at the last contact were censored. Median follow-up time was estimated using the reverse Kaplan–Meier method, and median OS was estimated using the Kaplan–Meier method. The median follow-up time was 28 months in the training cohort and 47 months in the held-out test set; the median OS for the entire cohort was 23 months. All enrolled patients underwent standardized postoperative follow-up: assessments every 3–4 months in the first year, every 6 months in years 2–5, and annually thereafter. Follow-up included: (1) systematic evaluation of clinical symptoms; (2) laboratory tests (complete blood count, liver and kidney function, etc.); and (3) imaging review (CT or MRI). The follow-up period ranged from 1 to 183 months. The cutoff date for follow-up was April 1, 2025. All data were verified for accuracy. The reporting of survival endpoints, event counts, and time-dependent performance metrics followed contemporary guidance for prognostic model evaluation and survival analysis (19, 20).

### CT imaging protocol

CECT images were acquired using six high-performance CT scanners: Canon Aquilion ONE, GE Optima CT, GE Revolution CT, Siemens Sensation Cardiac 64, Siemens SOMATOM Perspective, and Siemens SOMATOM Definition Flash. Scanning parameters were as follows: tube current modulation between 240 and 320 mAs, fixed tube voltage of 120 kVp, pitch of 1.375, reconstruction matrix of 512 × 512 pixels, and slice thickness of 5 mm. All patients received intravenous non-ionic contrast agent (Ultravist 370, Bayer Healthcare) at a flow rate of 3 mL/s and a dose of 1.5 mL/kg body weight. Three-phase enhanced scanning was performed: arterial phase (AP, 30 s), venous phase (VP, 60–70 s), and delayed phase (DP, 180 s). All images were exported in DICOM format from the PACS for subsequent radiomic analysis. Scanner distribution is detailed in **Supplementary Table 1**.

### Tumor segmentation and radiomic feature extraction

Tumor segmentation and feature extraction were performed following a standardized workflow. One radiologist (7 years of experience in abdominal imaging) and one urologist (15 years of experience in urologic oncology), both blinded to pathological results, manually delineated the region of interest (ROI) slice by slice on arterial, venous, and delayed phase CT images using ITK-SNAP (v4.2.0). The focus was on morphological characteristics and boundary definition, resulting in a 3D volume of interest (VOI). ROIs were confined to the primary tumor only, excluding metastatic lymph nodes or distant lesions even in N1 or M1 cases. Discrepancies were arbitrated by a third senior urologist (35 years of experience).

Features were extracted using the pyradiomics toolbox (Python 3.7) in compliance with the Image Biomarker Standardisation Initiative (IBSI) (21). Extracted features included first-order statistics, shape-based features, GLCM, GLDM, GLRLM, GLSZM, NGTDM, and wavelet features. A total of 854 radiomic features were obtained per patient. To mitigate scanner-related variations, all images were spatially normalized and resampled to a uniform voxel size of 1×1×1 mm³ using cubic spline interpolation (22).

Reliability was assessed by inter- and intra-observer reproducibility. Thirty cases were randomly selected from the training cohort only and independently segmented by two blinded urologists; the same physician repeated the segmentation after 4 weeks. The intraclass correlation coefficient (ICC) was calculated using a two-way random-effects model for absolute agreement (ICC(2, 1)). Features with an ICC > 0.750 were retained. This reproducibility assessment was performed without using outcome data from the held-out test set, and the 30 cases were not used for external validation. The approach to feature reproducibility and selection was informed by recent machine learning-based prognostic studies in oncology (23, 24).

### Feature selection and radiomics model construction

A standardized pipeline was used for feature selection and modeling. First, Z-score normalization was applied to all features using parameters derived solely from the training set to prevent data leakage.

A multistage feature selection strategy was employed within the training cohort: (1) features with ICC > 0.750 were retained; (2) univariable Cox regression identified prognostically relevant features; (3) models were built separately for AP, VP, and DP phases, as well as their combinations (AP+VP, AP+DP, VP+DP, AP+VP+DP). All phase combinations were evaluated using the same modeling pipeline and repeated 5-fold cross-validation in the training cohort. The performance of the AP+DP and VP+DP combinations is reported in the Results and in **Supplementary Table 2**. The final model was selected based on the highest mean cross-validation C-index, not on the held-out test set. The potential value of multiphase radiomics and the implications of omitting the delayed phase were interpreted in light of recent multiphase imaging and radiomics studies (24, 25).

Nine ML algorithms were compared within the training cohort: SuperPC, stepwise Cox, RSF, CoxBoost, Lasso, Ridge, Enet, survival-SVM, and plsRcox. Models with ≥2 features were considered valid. The optimal radiomics score (Rad-score) was selected based on the highest average C-index across repeated 5-fold cross-validation performed exclusively in the training cohort. Because more than 40 algorithm and phase combinations were evaluated, we explicitly prespecified that the held-out test set would be used only once, after model selection, to avoid data leakage. To quantify the risk of selection bias, we report the distribution of held-out test C-index values across all candidate AP+VP combinations and the ranking of the selected model within this distribution (**Supplementary Table 3**). We did not use the held-out test set to guide model choice. The potential for selection bias and the limitations of data-driven model selection were interpreted in accordance with recent frameworks for machine learning model selection and risk stratification (26, 27).

For the final Lasso + RSF model based on AP+VP features, the full list of retained features, their Lasso coefficients, RSF variable importance, and mean absolute Shapley additive explanations (SHAP) contributions is provided in **Supplementary Table 4**. Feature selection stability was assessed across repeated 5-fold cross-validation. The selection frequency of each retained feature is reported in **Supplementary Table 5**. The most stable feature (wavelet_LHH_glcm_ClusterShade_AP) was retained in 82% of the cross-validation folds, whereas the remaining key features showed moderate selection frequencies (6%–66%), reflecting the well-known instability of radiomic feature selection in small cohorts. Recent oncology machine learning studies have similarly emphasized transparent reporting of retained features, model coefficients, and feature stability (23, 24).

### Clinical and combined model development

Three predictive models were constructed: (1) a clinical model using multivariable Cox regression with independent prognostic factors identified from univariable analysis in the training cohort; (2) a radiomics model based on the optimal Rad-score; and (3) a combined model integrating the Rad-score and clinical predictors via multivariable Cox regression. Variables that were statistically significant in univariable analysis were subsequently included in a multivariable Cox regression model using the backward likelihood ratio method in the training cohort. The clinical risk score, AP+VP Rad-score, and combined model were constructed using training-cohort coefficients only. Model development adhered to the TRIPOD guidelines and was framed according to recent guidance on clinical prediction models and population-based prognostic modeling (26, 28).

### Model evaluation and interpretation

After the final combined model was locked, it was applied once to the held-out test set without further modification. Repeated 5-fold cross-validation and bootstrap validation (1000 resamples) were performed within the training cohort to estimate optimism and correct the C-index. The held-out test set was used only for final assessment of discrimination, calibration, and clinical utility. The combined model (AP+VP Rad-score plus clinical factors) was compared with the clinical model alone using bootstrap-derived differences in C-index.

A prognostic nomogram was developed to estimate individual survival probability. Time-dependent receiver operating characteristic (ROC) curves at 1, 2, and 3 years were used to assess discriminative ability. Calibration curves were plotted using bootstrap resampling (1000 repetitions). Decision curve analysis (DCA) was used to evaluate clinical utility across threshold probabilities.

The median nomogram score derived from the training cohort was used as the cutoff for risk stratification and applied to both cohorts. Kaplan-Meier curves and log-rank tests were used to compare survival between groups. SHAP analysis was performed to interpret feature contributions. SHAP values were computed on the training cohort using the final locked model; the held-out test set was reserved for performance evaluation and was not used for SHAP-based interpretation. The SHAP analysis was exploratory and *post hoc* rather than prespecified. For the radiomics-only model, SHAP values were derived from the final Lasso + RSF model; for the combined model, SHAP values were derived from the final multivariable Cox model incorporating the AP+VP Rad-score, N stage, M stage, and Ki-67 index. Positive SHAP values indicate contributions toward a higher predicted risk of death. The reporting and interpretation of SHAP results were guided by contemporary frameworks for interpretable machine learning in clinical prediction (27, 29).

### Statistical analysis

Analyses were performed using IBM SPSS Statistics 26.0 and R 4.4.3. All tests were two-sided, with α = 0.05. Categorical variables are presented as frequencies (%), and compared using Pearson’s χ² or Fisher’s exact test. Continuous variables are expressed as mean ± SD or median (IQR) according to normality (Shapiro-Wilk test) and compared using the t-test or Mann–Whitney U test. Survival analyses were performed using the Kaplan–Meier method and the log-rank test. Prognostic factors were identified using univariable and multivariable Cox regression; results are reported as hazard ratios (HRs) with 95% confidence intervals (CIs). The interpretation of biomarker-based prognostic estimates followed contemporary frameworks for biomarker validation and risk stratification in oncology (29, 30).

## Results

### Baseline clinical characteristics of patients

According to the predefined inclusion and exclusion criteria, 113 patients with sRCC were included and allocated to a training cohort (n = 75) and a held-out test set (n = 38). The median follow-up time, estimated using the reverse Kaplan–Meier method, was 28 months in the training cohort and 47 months in the held-out test set; the median OS for the entire cohort was 23 months (follow-up range: 1–183 months). During follow-up, 54 deaths were recorded: 31 in the training cohort and 23 in the held-out test set. Kaplan–Meier analysis revealed no significant difference in OS between the training and held-out test cohorts (P = 0.208). The numbers of patients at risk and events within 1, 2, and 3 years are summarized in **Supplementary Table 6**. In the training cohort, the numbers at risk at 1, 2, and 3 years were 54, 37, and 24, with 17, 20, and 26 events within these intervals, respectively. In the held-out test set, the corresponding numbers at risk were 25, 19, and 17, with 10, 15, and 16 events, respectively. Demographic and clinicopathological characteristics are summarized in **Table 1**. Most baseline characteristics were well balanced between the two cohorts (P > 0.05), indicating comparability. However, GLR differed significantly between the training and held-out test cohorts (P = 0.029), with a higher GLR in the training cohort. Because GLR was not retained as an independent predictor in the final multivariable model, this imbalance is unlikely to materially affect the main findings; nevertheless, it should be considered when interpreting the held-out test results.

**Table 1:** Demographic and clinical characteristics of patients in the training cohort and held-out test set.

Characteristics	Training cohort (n=75)	Held-out test set (n=38)	P-value
Age (years)	55.89 ± 11.80	60.00 ± 10.37	0.545
Gender			0.405
Female	20 (26.67%)	13 (34.21%)	
Male	55 (73.33%)	25 (65.79%)	
BMI	24.42 ± 3.72	24.25 ± 3.20	0.343
Hypertension			0.535
Yes	29 (38.67%)	17 (44.74%)	
No	46 (61.33%)	21 (55.26%)	
Diabetes Mellitus			0.459
Yes	14 (18.67%)	5 (13.16%)	
No	61 (81.33%)	33 (86.84%)	
Albumin	39.12 ± 6.22	39.03 ± 7.35	0.364
Alkaline Phosphatase	103.94 ± 64.84	99.84 ± 83.31	0.424
Cholesterol	4.48 ± 1.18	4.38 ± 1.10	0.524
LDH	212.70 ± 107.60	230.08 ± 220.82	0.211
Urea	5.51 ± 2.05	5.56 ± 1.75	0.432
Creatinine	86.42 ± 80.47	77.71 ± 20.65	0.272
Glucose	6.36 ± 2.94	5.78 ± 1.43	0.058
Fibrinogen	4.68 ± 1.47	4.72 ± 1.39	0.503
T stage			0.333
T1	21 (28.00%)	11 (28.95%)	
T2	15 (20.00%)	3 (7.90%)	
T3	26 (34.67%)	18 (47.37%)	
T4	13 (17.33%)	6 (15.78%)	
N stage			0.926
N0	50 (66.67%)	25 (65.79%)	
N1	25 (33.33%)	13 (34.21%)	
M stage			0.640
M0	48 (64.00%)	26 (68.42%)	
M1	27 (36.00%)	12 (31.58%)	
Tumor Size	7.84 ± 3.45	7.41 ± 3.24	0.835
Ki-67	0.30 ± 0.20	0.29 ± 0.22	0.498
SII	1217.88 ± 1125.00	1109.02 ± 904.19	0.637
PNI	47.63 ± 7.80	47.54 ± 7.48	0.671
GLR	4.58 ± 3.38	3.79 ± 1.60	0.029

PNI, albumin + 5 × lymphocyte; SII, neutrophil × platelet/lymphocyte; GLR, Glucose/lymphocyte.

### Feature selection, prognostic model construction, and interpretation

A total of 854 quantifiable radiomic features were extracted from each phase (AP, VP, and DP) of the ROI. After inter- and intra-observer consistency analysis (ICC > 0.750), 781 (AP), 749 (VP), and 710 (DP) features were retained for further analysis. In the training cohort, univariable Cox regression identified 98 (AP), 80 (VP), and 58 (DP) features significantly associated with prognosis (P < 0.05). We systematically compared more than 40 algorithm and phase combinations (**Supplementary Table 2**). The Lasso + RSF model based on AP+VP features achieved the highest average C-index across repeated 5-fold cross-validation and was therefore selected as the final radiomics model. In the held-out test set, the selected model achieved a C-index of 0.735 (95% CI: 0.604–0.852), compared with 0.892 (95% CI: 0.837–0.945) in the training cohort (**Figure 2A**).

**Figure 2:**
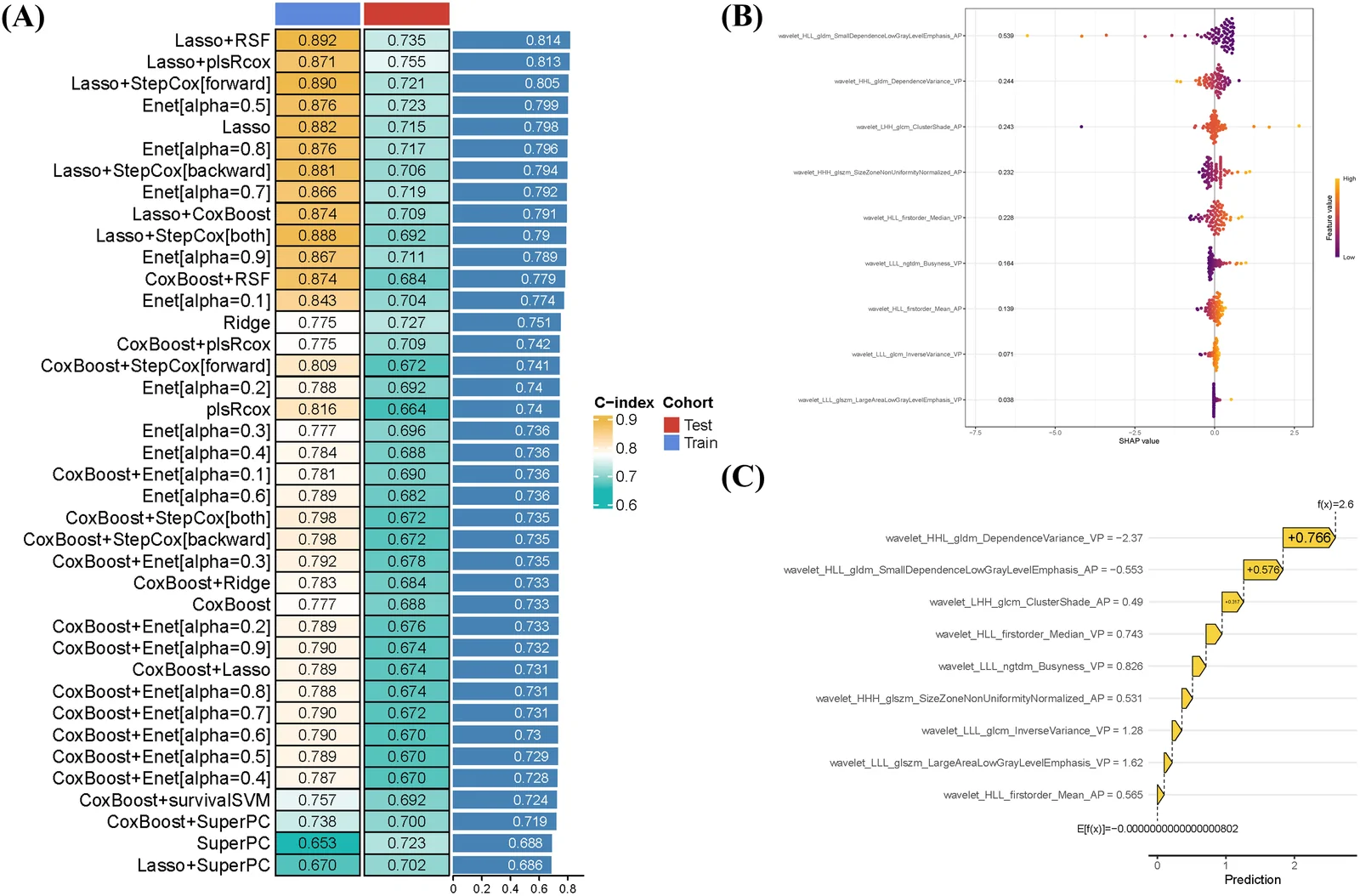
Prognostic prediction model performance and SHAP interpretability analysis based on combined arterial phase (AP) and venous phase (VP) radiomic features. **(A)** Predictive performance of the Lasso-random survival forest (RSF) model constructed using combined AP+VP features. The heatmap color gradient (green to yellow) represents increasing C-index values, with the training and validation sets achieving C-indices of 0.892 and 0.735, respectively. **(B)** Global SHAP summary plot demonstrating the contribution distribution of the 9 most influential features. Dark purple and yellow hues indicate negative and positive impacts on prediction outcomes, respectively. **(C)** Waterfall plot illustrating an individual high-risk patient’s prediction, demonstrating the model’s capability for accurate risk stratification. Both the baseline predicted probability (E[f(x)] = −8.02×10^−17^) and the individual predicted value (f(x) = 2.6) are annotated.

To contextualize this performance, we examined the distribution of held-out test C-index values across the 39 AP+VP algorithm combinations evaluated. The test C-index ranged from 0.664 to 0.755, with a median of 0.692 (IQR: 0.674–0.711). The selected Lasso + RSF model ranked second (C-index: 0.735), corresponding approximately to the 97th percentile of this distribution. The highest test C-index was observed for Lasso + plsRcox (0.755), which differed from the selected model by only 0.020. This difference is well within the expected variability given the small held-out test set (n = 38; 23 deaths) and the wide 95% CI of the C-index (0.612–0.846). These findings suggest that the apparent superiority of the selected model over alternative combinations should be interpreted cautiously, as it may partly reflect random variation (26, 27).

As a *post hoc* exploratory comparison, we examined whether a simple average of the top-performing AP+VP models might provide more robust performance. Because of the small held-out test set and the limited number of events, formal ensemble averaging was not sufficiently powered in the present study. Nevertheless, the narrow spread of test C-index values among the top-ranked models (0.735–0.755) suggests that several modeling strategies performed similarly, and that prespecifying a single modeling strategy or averaging top-performing models may be preferable in future larger studies (26, 27).

The held-out test set was not used during feature selection, algorithm comparison, or model tuning and was evaluated only after the final model was locked. Time-dependent ROC curve analysis showed that the AUC values for predicting 1-, 2-, and 3-year OS in the held-out test set were 0.791, 0.758, and 0.729, respectively (**Supplementary Figure 1**).

Interpretability of the Lasso + RSF model was further analyzed using SHAP (Shapley Additive Explanations). The global SHAP beeswarm plot (**Figure 2B**) illustrates the distribution of SHAP values for the nine key features retained in the final Lasso + RSF model. Seven of these features contributed positively to predicting survival risk, whereas two features—wavelet_HLL_gldm_SmallDependenceLowGrayLevelEmphasis_AP and wavelet_HHL_gldm_DependenceVariance_VP—showed negative contributions, suggesting that higher values of these two texture features may be associated with a relatively favorable prognosis. The specific features, their Lasso coefficients, RSF variable importance, and mean absolute SHAP values are listed in **Supplementary Table 4**. The stability of feature selection across repeated 5-fold cross-validation is summarized in **Supplementary Table 5**; the most stable feature was retained in 82% of folds, whereas the remaining key features showed moderate selection frequencies (6%–66%), consistent with the known instability of radiomic feature selection in small cohorts. Individual prediction visualization (**Figure 2C**) demonstrated a typical high-risk patient, in whom the baseline prediction probability (E[f(x)] = −8.02 × 10^−17^) represents the model threshold and the individual prediction value (f(x) = 2.6) reflects the final risk assessment.

### Construction of clinical and combined models

Univariable Cox regression identified several clinical features significantly associated with OS in patients with sRCC (**Table 2**). BMI, alkaline phosphatase, fibrinogen, TNM stage, tumor size, Ki-67 index, and systemic immune-inflammation index (SII) were significantly correlated with OS in the training cohort (P < 0.05). Variables that were statistically significant in univariable analysis were subsequently included in a multivariable Cox regression model using the backward likelihood ratio method. Multivariable analysis confirmed that N stage (HR = 3.210, 95% CI: 1.376–7.490; P < 0.01), M stage (HR = 2.353, 95% CI: 1.074–5.153; P = 0.032), and Ki-67 index (HR = 5.938, 95% CI: 1.298–27.164; P = 0.022) were independent prognostic factors for OS in patients with sRCC (**Table 2**). Ki-67 was analyzed as a continuous variable on the proportional scale (0–1). The 95% CI for Ki-67 was wide, indicating limited precision of the effect estimate. This imprecision likely reflects the relatively small number of events, the continuous scaling of Ki-67, and the single-center retrospective design. Therefore, although Ki-67 was statistically significantly associated with OS, this result should be interpreted with caution and considered hypothesis-generating rather than definitive. Based on these factors, a clinical prognostic model was constructed, and a clinical risk score was computed for each patient. Finally, a combined prognostic model was developed by integrating the AP+VP radiomic risk score (AP+VP-Radscore) with clinical variables including N stage, M stage, and Ki-67 index.

**Table 2:** Univariate and multivariate Cox regression analyses of clinical characteristics in the training cohort using backward LR (likelihood ratio) selection.

Variables	Univariate analysis		P value	Multivariate analysis		P value
	HR	95% CI		HR	95% CI	
Age	0.992	0.963-1.021	0.578			
Sex						
Female	Ref					
Male	1.473	0.630-3.445	0.372			
BMI	0.909	0.830-0.996	0.040			
Hypertension						
No	Ref					
Yes	0.728	0.345-1.534	0.403			
Diabetes Mellitus						
No	Ref					
Yes	0.585	0.204-1.675	0.318			
Albumin	0.955	0.902-1.011	0.111			
Alkaline Phosphatase	1.005	1.000-1.009	0.033			
Cholesterol	0.858	0.623-1.183	0.350			
LDH	1.001	0.998-1.004	0.517			
Urea	0.905	0.749-1.094	0.303			
Creatinine	1.001	0.998-1.003	0.698			
Glucose	1.068	0.974-1.204	0.285			
Fibrinogen	1.347	1.086-1.670	0.007			
T stage						
T1	Ref					
T2	2.366	0.608-9.214	0.214			
T3	2.986	0.832-10.724	0.093			
T4	5.744	1.570-21.019	0.008			
N stage						
N0	Ref			Ref		
N1	5.081	2.378-10.854	<0.001	3.210	1.376-7.490	<0.01
M stage						
M0	Ref			Ref		
M1	3.333	1.624-6.844	<0.001	2.353	1.074-5.153	0.032
Tumor size	1.111	1.016-1.216	0.021			
Ki-67	10.154	2.199-46.892	<0.01	5.938	1.298-27.164	0.022
SII	1.000	1.000-1.001	<0.01			
GLR	1.063	0.969-1.167	0.197			
PNI	0.960	0.920-1.002	0.060			

PNI, albumin + 5 × lymphocyte; SII, neutrophil × platelet/lymphocyte; GLR, Glucose/lymphocyte.

### Development and validation of the combined prognostic nomogram

Based on the clinical prognostic factors and the AP+VP radiomic risk score (AP+VP-Radscore), a nomogram was developed to predict 1-, 2-, and 3-year OS in patients with sRCC (**Figure 3A**). The nomogram is applied as follows: (1) locate the score for each predictive variable on the top point scale; (2) project each score vertically to the Points axis; (3) sum all points to obtain the Total Points; and (4) determine the predicted 1-, 2-, and 3-year survival probabilities on the bottom survival probability axis corresponding to the Total Points.

**Figure 3:**
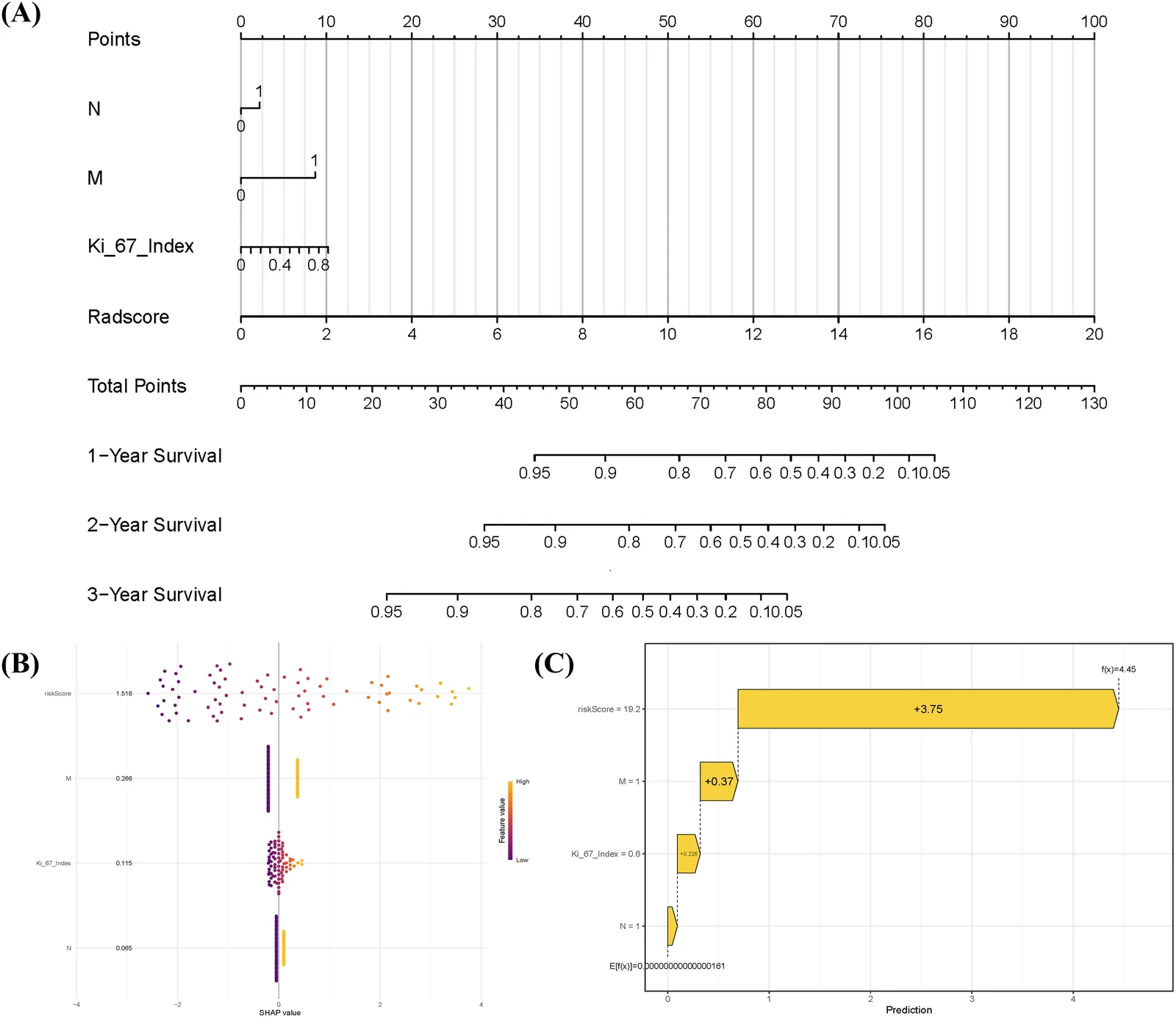
Nomogram and SHAP interpretability analysis of the combined prognostic model. **(A)** Nomogram for predicting overall survival (OS) in sRCC patients using the combined model. **(B)** Global SHAP summary plot illustrating the contribution distribution of the four most influential features, where deep purple and yellow hues represent negative and positive impacts on prediction outcomes, respectively. **(C)** Waterfall plot demonstrating individualized risk prediction for a representative high-risk patient, illustrating how the model combines feature contributions for a representative patient. Both baseline predicted probability (E[f(x)] = 1.61×10^−15^) and individual predicted value (f(x) = 4.45) are annotated.

SHAP analysis was applied to interpret the combined model. The global SHAP beeswarm plot (**Figure 3B**) showed that all four key predictive features had positive contributions (SHAP values > 0), indicating a significant association with poor prognosis. Individual prediction visualization (**Figure 3C**) illustrated the prediction for a representative high-risk patient, in whom the baseline prediction risk (E[f(x)] = 1.610 × 10^−15^) represents the model threshold and the individual prediction risk (f(x) = 4.450) was significantly higher, consistent with clinical observations.

### Predictive performance and clinical validation of the combined prognostic model

As shown in **Table 3**, the combined model showed numerically higher discriminative ability for predicting OS in patients with sRCC than the clinical model, but this improvement was not statistically significant. In the held-out test set, the C-index was 0.737 (95% CI: 0.612–0.846) for the combined model, 0.735 (95% CI: 0.604–0.852) for the AP+VP radiomics model, and 0.615 (95% CI: 0.488–0.756) for the clinical model; the combined model was therefore comparable to the radiomics-only model.

**Table 3:** Discriminative performance of clinical, radiomics, and combined models for predicting overall survival in sRCC patients.

Model	C-Index	AUC		
		1-Year OS	2-Year OS	3-Year OS
Training cohort					
Clinical model	0.805	0.880	0.827	0.845
Radiomics model	0.892	0.926	0.896	0.944
Combined model	0.910	0.948	0.923	0.962
Held-out test set					
Clinical model	0.615	0.647	0.671	0.712
Radiomics model	0.735	0.791	0.758	0.729
Combined model	0.737	0.833	0.780	0.749

After repeated cross-validation, the mean C-indices of the clinical, radiomic, and combined models in the training cohort were 0.787 (± 0.090), 0.903 (± 0.058), and 0.898 (± 0.058), respectively. After bootstrap optimism correction, the C-indices were 0.785, 0.890, and 0.896, respectively. In the held-out test set, the C-indices were 0.615, 0.735, and 0.737, respectively (**Supplementary Table 7**).

To quantitatively compare the performance of the combined and clinical models in the held-out test set, bootstrap resampling (1000 repetitions) was used to assess the difference in C-indices. The bootstrap-estimated difference (clinical minus combined) was −0.122 (95% CI: −0.269 to 0.042; P = 0.115), corresponding to a numerical advantage of 0.122 for the combined model; however, this difference was not statistically significant.

Time-dependent ROC analysis further evaluated the predictive accuracy of the combined model. In the training cohort, the AUC values for predicting 1-, 2-, and 3-year OS were 0.948, 0.923, and 0.962, respectively (**Figure 4A**); the corresponding AUC values in the held-out test set were 0.833, 0.780, and 0.749 (**Figure 4B**). These time-dependent AUC estimates should be interpreted in the context of the numbers of events available at each time point. In the held-out test set, the 1-, 2-, and 3-year AUC values were based on 10, 15, and 16 events, respectively, with 25, 19, and 17 patients at risk (**Supplementary Table 6**). The 3-year AUC (0.749) was therefore derived from a relatively small number of events, which may lead to imprecise and potentially unstable estimates. Bootstrap tests showed that at 1 year, the AUC difference between the combined and clinical models was −0.186 (95% CI: −0.435 to 0.056; P = 0.140); at 2 years, it was −0.109 (95% CI: −0.353 to 0.118; P = 0.363); and at 3 years, it was −0.037 (95% CI: −0.258 to 0.184; P = 0.745). Comparisons of ROC curves at different time points in the validation set are shown in **Supplementary Figure 2**. Although the combined model consistently showed numerically higher AUC values than the clinical model across all time points, these differences were not statistically significant. Given the limited number of events, particularly at 3 years, these comparisons should be considered exploratory and interpreted with caution (19, 20).

**Figure 4:**
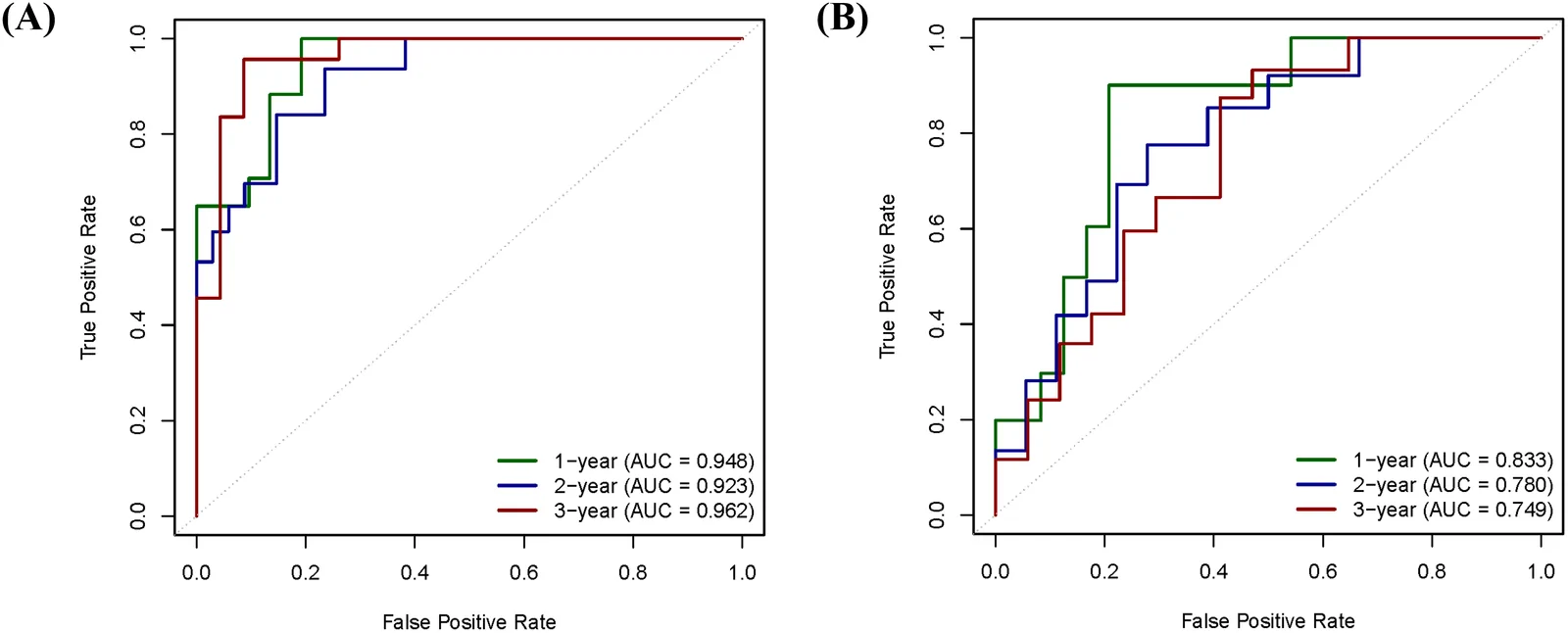
Time-dependent receiver operating characteristic (ROC) curves for survival prediction based on nomogram risk scores. **(A)** Overall survival (OS) prediction in the training cohort. **(B)** OS prediction in the held-out test set.

Calibration analysis showed that the combined model had good calibration in both the training and held-out test cohorts, with predicted survival probabilities closely aligning with observed outcomes (**Figures 5A, B**). DCA demonstrated favorable clinical utility of the combined model for predicting 1-, 2-, and 3-year survival. Across a wide range of threshold probabilities, the combined model provided higher net benefit than the “treat all” or “treat none” strategies (**Figure 6**). Based on the median nomogram score, patients were stratified into high- and low-score groups. Kaplan–Meier survival analysis confirmed that patients in the high-score group had significantly shorter OS than those in the low-score group in both the training and held-out test cohorts (log-rank P < 0.05) (**Figure 7**), supporting the clinical applicability of this scoring system.

**Figure 5:**
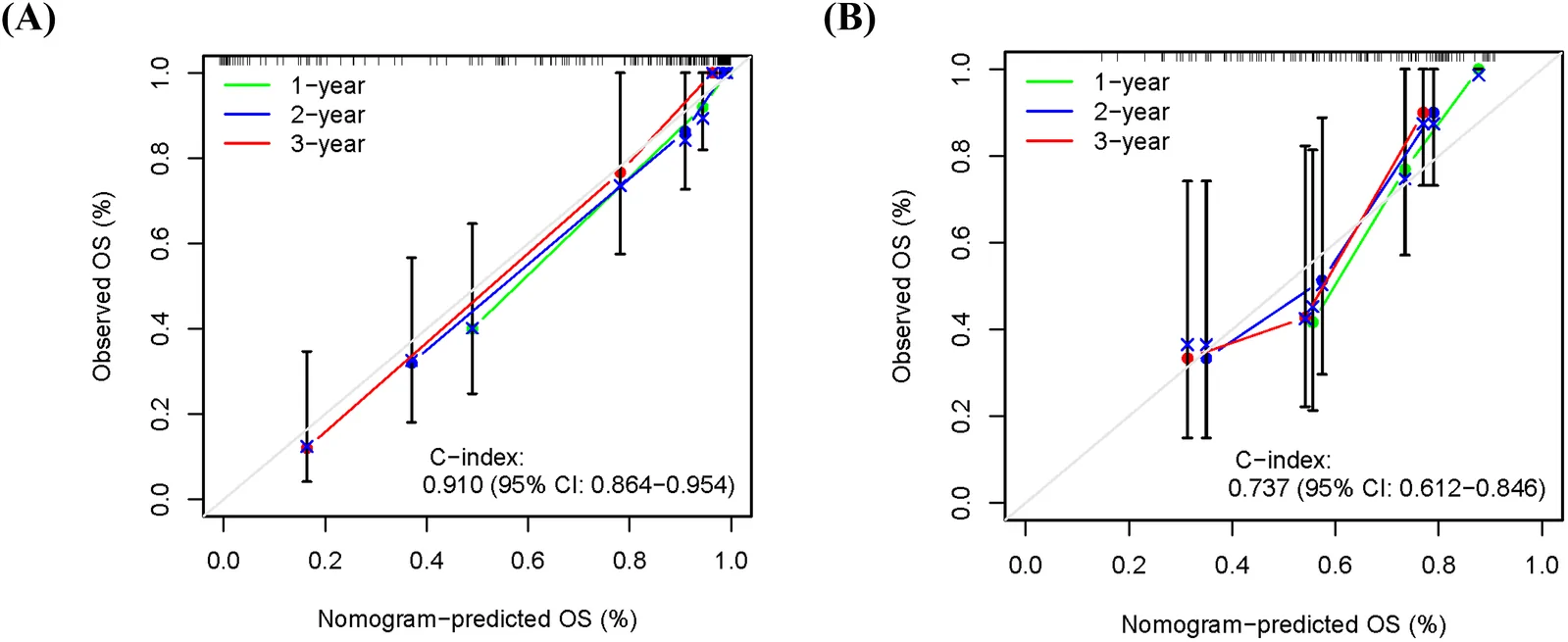
Calibration curves assessing the predictive accuracy of the nomogram for overall survival (OS). **(A)** Calibration plot for OS prediction in the training cohort. **(B)** Calibration plot for OS prediction in the held-out test set.

**Figure 6:**
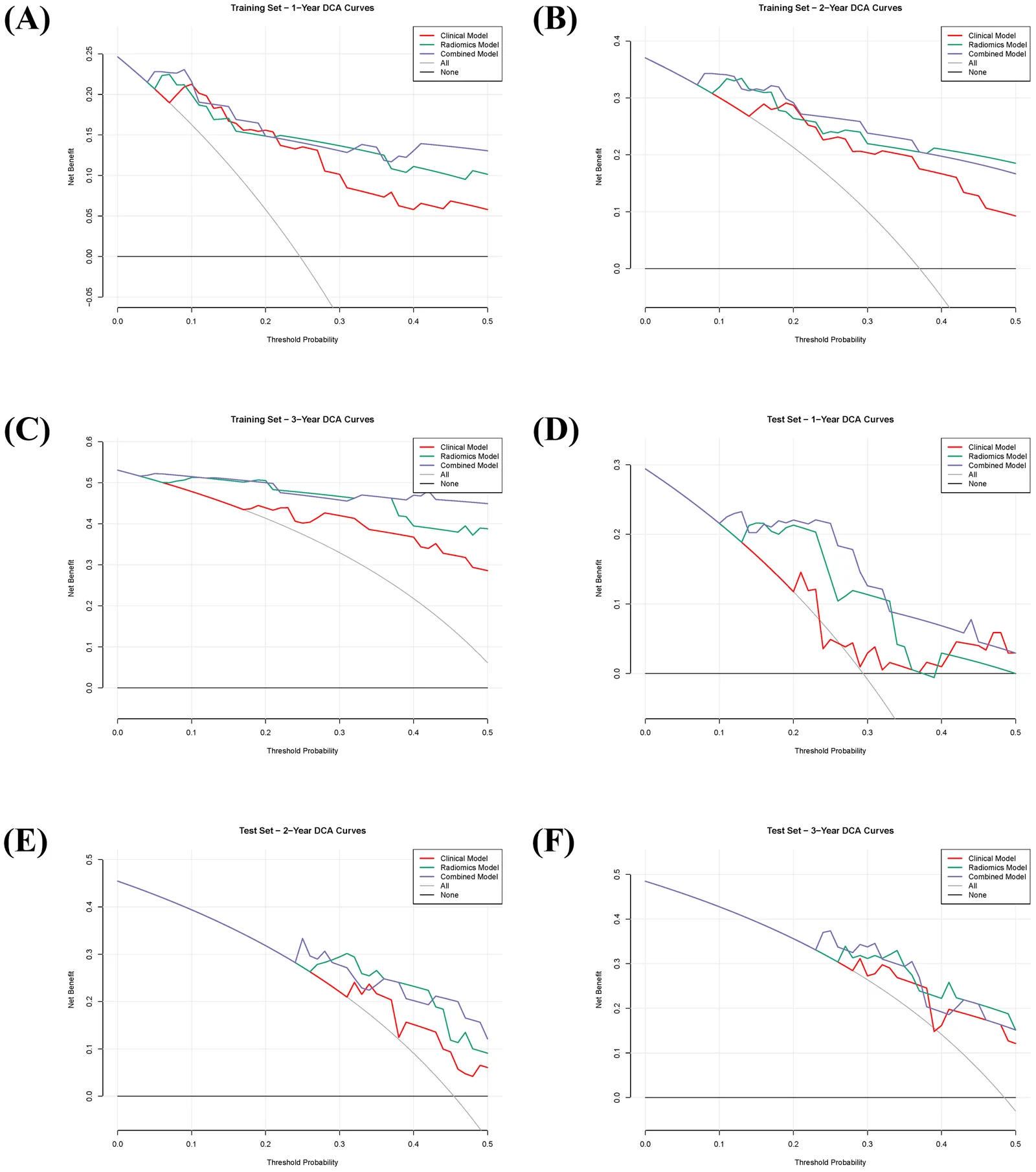
Decision curve analysis (DCA) for evaluating the clinical net benefit of different prognostic models in both training and validation cohorts at 1-, 2-, and 3-year time points. The DCA curves compare the standardized net benefit across a range of threshold probabilities for the clinical model, radiomics model, and combined model. The net benefits of the “treat all” (green line) and “treat none” (gray line) strategies are shown as references. **(A)** DCA for 1-year overall survival prediction in the training cohort. **(B)** DCA for 2-year overall survival prediction in the training cohort. **(C)** DCA for 3-year overall survival prediction in the training cohort. **(D)** DCA for 1-year overall survival prediction in the held-out test set. **(E)** DCA for 2-year overall survival prediction in the held-out test set. **(F)** DCA for 3-year overall survival prediction in the held-out test set.

**Figure 7:**
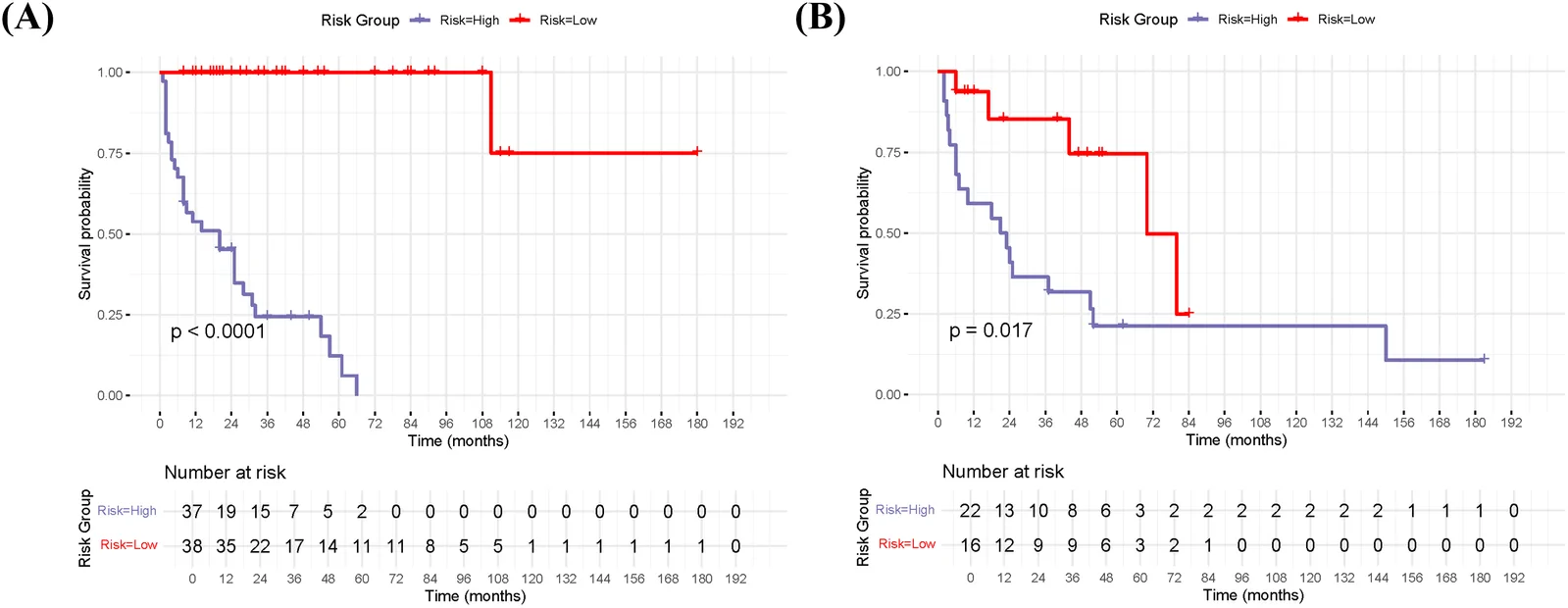
Risk stratification based on median nomogram risk scores. **(A)** Kaplan-Meier survival curves for the training cohort. **(B)** Kaplan-Meier survival curves for the held-out test set.

## Discussion

sRCC is a highly aggressive malignancy, accounting for approximately 20% of advanced RCC cases (31, 32). Clinical observations indicate that approximately 70% of patients with sRCC present with distant metastasis at diagnosis (33). Although the AJCC TNM staging system remains the gold standard for prognostic assessment, the substantial survival heterogeneity observed among patients within the same stage (34) underscores the limitations of relying solely on anatomical parameters for outcome prediction. In recent years, radiomics combined with ML has emerged as a promising approach for cancer prognosis. By enabling high-throughput extraction of CT image features, this technique permits quantitative assessment of tumor heterogeneity and facilitates the development of more accurate prognostic models through integration with clinical parameters (16, 18, 35). However, evidence systematically evaluating the efficacy of different ML algorithms in predicting sRCC prognosis remains scarce. To our knowledge, this study is the first to integrate multiphase CECT radiomic features with nine ML algorithms to develop and validate a prognostic prediction model for sRCC, providing a novel objective basis for clinical decision-making.

This study included 113 patients with pathologically confirmed sRCC. Through multiphase CECT radiomic analysis, we identified imaging features with significant prognostic value and constructed an optimal predictive model using Lasso regression combined with RSF. The combined model, incorporating clinical indicators and AP+VP radiomic features, exhibited numerically improved predictive performance compared with the clinical model. In the held-out test set, the model achieved a Harrell’s C-index of 0.737 (95% CI: 0.612–0.846) versus 0.615 (95% CI: 0.488–0.756) for the clinical-only model; however, this difference was not statistically significant (P = 0.115). Its performance was also comparable to that of the AP+VP radiomics-only model (C-index: 0.735; 95% CI: 0.604–0.852). The reporting of prediction model performance was guided by recent frameworks for prognostic model evaluation and nomogram reporting (19, 36).

The combined model developed in this study showed performance comparable to that of the radiomics-only model and numerically higher performance than the clinical model; however, the incremental value over radiomics alone was minimal in the held-out test set. Previous studies have shown that radiomic features can effectively reflect the biological and pathological characteristics of tumors (37–39). Several studies have successfully applied radiomic models to predict metastatic potential and International Society of Urological Pathology (ISUP) grade, confirming their prognostic value (38, 39). In the field of RCC prognosis, radiomics has shown considerable promise. Gao et al. (40) developed a nomogram integrating radiomic features and clinicopathological parameters to predict ccRCC prognosis (AUC = 0.768). He et al. (41) constructed a prognostic model based on a radiomic score, achieving AUCs of 0.826, 0.805, and 0.760 at 12, 36, and 60 months, respectively. Notably, all features selected in the AP+VP radiomic model in our study were wavelet-based. Previous studies have confirmed that wavelet transform and Laplacian of Gaussian (LoG) filtering can effectively quantify tumor heterogeneity (42). Wavelet transform offers unique advantages: (1) it can extract local and high-dimensional information beyond human visual perception; (2) it exhibits strong noise resistance at large scales; and (3) it precisely captures fine image details at small scales, achieving an optimal balance between noise suppression and edge feature extraction (42, 43). Several studies support the value of wavelet features in prognostic evaluation. Jiao et al. (44) found that wavelet features were significantly associated with poor prognosis in patients with early-stage lung cancer. By integrating clinical and radiomic parameters, our study did not significantly improve predictive performance beyond radiomics alone. The combined model achieved C-index values comparable to those of the radiomics-only model in both the training and validation sets, supporting its potential discriminative ability and clinical applicability as an objective tool for survival analysis in sRCC.

Multivariable Cox regression confirmed that N stage (HR = 3.210, 95% CI: 1.376–7.490; P < 0.01), M stage (HR = 2.353, 95% CI: 1.074–5.153; P = 0.032), and Ki-67 index (HR = 5.938, 95% CI: 1.298–27.164; P = 0.022) were independent prognostic factors for OS in patients with sRCC. These findings align with previous studies indicating that clinicopathological features such as TNM stage and histological grade are significantly associated with long-term cancer outcomes (39, 41, 45). The Ki-67 nuclear antigen, a key marker of cellular proliferation, plays an important role in assessing tumor heterogeneity and aggressiveness. By regulating cell cycle progression, this protein promotes tumor proliferation (46) and has been associated with poor prognosis in various malignancies (47, 48). In our study, Ki-67 was measured as the percentage of tumor cells with positive nuclear staining and was modeled as a continuous variable. The association between Ki-67 and OS was statistically significant, but the 95% CI was wide (1.298–27.164), indicating substantial uncertainty around the effect estimate. This finding is consistent with the direction of the association reported by Wang et al. (49) in sRCC, but the precision of our estimate is limited. The prognostic value of Ki-67 in sRCC should therefore be further evaluated using standardized immunohistochemical protocols, prespecified cutoffs, and larger multicenter cohorts. Recent work on biomarker validation and risk stratification in oncology emphasizes the importance of transparent reporting of assay methodology, cutoff selection, and external validation (29, 30). Particularly noteworthy is that multiple studies have reported that high Ki-67 expression is significantly associated with increased risk of recurrence, shorter OS, and shorter disease-free survival (DFS) in patients with ccRCC (50). Based on these findings, we constructed a multiparameter predictive model by integrating clinicopathological factors with significant prognostic value (including the Ki-67 index) and radiomic features. This comprehensive analytical approach is expected to provide more accurate prognostic assessment for patients with sRCC and to lay the foundation for future research.

Despite the clinically relevant findings, this study has several limitations that should be acknowledged. First, the single-center retrospective design and limited sample size (n = 113) may introduce selection bias and reduce statistical power; future validation through multicenter, large-sample prospective studies is warranted. Second, retrospective data collection may lead to information bias, with incomplete availability of some clinical parameters. Known prognostic factors such as detailed systemic treatment regimens and the percentage of sarcomatoid components were not systematically included. Although key variables were adjusted for in multivariable analysis, residual confounding cannot be entirely excluded. Third, radiomic analysis was based only on ROIs drawn on the primary tumor, without including features from metastatic lesions or peritumoral regions. Additionally, the feature set did not incorporate LoG filter transformations, which may limit the model’s ability to fully capture tumor heterogeneity. Fourth, although rigorous internal validation strategies such as repeated cross-validation were employed, the performance of the combined model in the held-out test set (C-index: 0.737) was lower than that in the training set (C-index: 0.898; bootstrap-corrected C-index: 0.896), and the difference from the clinical model did not reach statistical significance (P = 0.115). Moreover, the combined model was comparable to the radiomics-only model in the held-out test set, possibly due to the small validation sample size (n = 38). The lack of external multicenter validation limits assessment of the model’s generalizability.

Future work should involve prospective, multicenter, large-sample studies and external validation to enhance model robustness. Additionally, integrating imaging features from metastatic and peritumoral regions, incorporating multi-omics data (e.g., genomics, proteomics), and employing deep learning algorithms may help build a more comprehensive and accurate prognostic evaluation system.

## Conclusion

This study establishes an ML-based prognostic model integrating multiphase CT radiomics features (AP/VP) and clinicopathological factors (N stage, M stage, and Ki-67 index) for sRCC. The combined model showed numerically higher predictive accuracy than clinical parameters alone; however, this improvement was not statistically significant, and its performance was comparable to that of the AP+VP radiomics model. As a noninvasive tool, it may provide individualized survival stratification to guide clinical decision-making, with the SHAP framework enhancing interpretability. Although the retrospective design limits generalizability, these findings support radiomics-ML integration as a potential strategy for refining prognosis assessment in sRCC.

## Data Availability

The original contributions presented in the study are included in the article/**Supplementary Material**. Further inquiries can be directed to the corresponding author.

## Supplementary material

Supplementary Table 1 Distribution of CT scanners and corresponding case numbers in this study.

Supplementary Table 2 Performance of algorithm and phase combinations in the training cohort and held-out test set.

Supplementary Table 3 Distribution of held-out test C-index across AP+VP algorithm combinations.

Supplementary Table 4 Retained radiomic features in the final Lasso + RSF model.

Supplementary Table 5 Stability of feature selection across repeated 5-fold cross-validation.

Supplementary Table 6 Numbers at risk and events at 1, 2, and 3 years in the training cohort and held-out test set.

Supplementary Table 7 Predictive performance of different prognostic models in the training cohort and held-out test set.
