# Supplementary material for: Development and validation of a multiphase CT radiomics-based machine learning model for prognostic prediction in sarcomatoid renal cell carcinoma
Source: Front Oncol. 2026 Oct 30;16:1982796. doi: 10.3389/fonc.2026.1982796 (PMC13759397; doi:10.3389/fonc.2026.1982796)
Supplement: Supplementary file 1 [file SupplementaryFile1.docx]

*Supplementary Figure 1. Time-dependent ROC curves of the optimal radiomics model (Lasso + RSF based on AP + VP features) for predicting 1-year, 2-year, and 3-year overall survival in the held-out test set.*

*Supplementary Figure 2. Time-dependent ROC curves for predicting 1-, 2-, and 3-year overall survival in the held-out test set, comparing clinical, radiomics, and combined models.*
