# Supplementary material for: Development and validation of a multiphase CT radiomics-based machine learning model for prognostic prediction in sarcomatoid renal cell carcinoma
Source: Front Oncol. 2026 Oct 30;16:1982796. doi: 10.3389/fonc.2026.1982796 (PMC13759397; doi:10.3389/fonc.2026.1982796)
Supplement: Supplementary file 2 [file Table1.docx]

Supplementary Table 1: Distribution of CT scanners and corresponding case numbers in this study

| CT Scanner Model | Number of Cases |
| --- | --- |
| Aquilion ONE | 17 |
| Optima CT | 22 |
| Revolution CT | 23 |
| Sensation Cardiac 64 | 5 |
| SOMATOM Perspective | 15 |
| SOMATOM Definition Flash | 31 |
