# Supplementary material for: Development and validation of a multiphase CT radiomics-based machine learning model for prognostic prediction in sarcomatoid renal cell carcinoma
Source: Front Oncol. 2026 Oct 30;16:1982796. doi: 10.3389/fonc.2026.1982796 (PMC13759397; doi:10.3389/fonc.2026.1982796)
Supplement: Supplementary file 3 [file Table6.docx]

**Supplementary Table 6. Numbers at risk and events at 1, 2, and 3 years in the training cohort and held-out test set**

| Time point | Training cohort (n = 75) | Held-out test set (n = 38) |
| --- | --- | --- |
| **1 year** |  |  |
| Number at risk | 54 | 25 |
| Events within 1 year | 17 | 10 |
| **2 years** |  |  |
| Number at risk | 37 | 19 |
| Events within 2 years | 20 | 15 |
| **3 years** |  |  |
| Number at risk | 24 | 17 |
| Events within 3 years | 26 | 16 |
