# Supplementary material for: Development and validation of a multiphase CT radiomics-based machine learning model for prognostic prediction in sarcomatoid renal cell carcinoma
Source: Front Oncol. 2026 Oct 30;16:1982796. doi: 10.3389/fonc.2026.1982796 (PMC13759397; doi:10.3389/fonc.2026.1982796)
Supplement: Supplementary file 4 [file Table7.docx]

Supplementary Table 7. Predictive performance of different prognostic models in the training cohort and held-out test set

| Model | Original training C-index | Optimism | Optimism-corrected C-index | Held-out test C-index |
| --- | --- | --- | --- | --- |
| Clinical model | 0.805 | 0.020 | 0.785 | 0.615 |
| Radiomics model | 0.892 | 0.002 | 0.890 | 0.735 |
| Combined model | 0.910 | 0.014 | 0.896 | 0.737 |
